# 

**Published:** 1889-07-06

**Authors:** 


**PRICE ONE PENNY.**

No. 145, Vol. VI.—July 6th, 1889.

[Registered at the General Post Office as a Newspaper.]

Per Annum, 6s. 6d., post free.

Monthly Parts, 6d.; post free 7½d.

